# Reassessment of Lymphovascular Invasion and Its Subtypes as Predictors of Prognosis and Recurrence in Gastric Cancer Using an Enhanced Detection Method

**DOI:** 10.3390/cancers18071101

**Published:** 2026-03-28

**Authors:** Jingdong Liu, Changle Yang, Bosen Li, Zhaodong Sun, Dan Liu, Xinyou Liu, Hao Chen, Jie Sun, Haojie Li, Yihong Sun, Junjie Zhao, Xuefei Wang

**Affiliations:** 1 Department of Gastrointestinal Surgery, Zhongshan Hospital, Fudan University, Shanghai 200032, China; liu.jingdong@zs-hospital.sh.cn (J.L.); yangcl18@fudan.edu.cn (C.Y.); bsli17@fudan.edu.cn (B.L.); 17301050209@fudan.edu.cn (Z.S.); 22111210085@m.fudan.edu.cn (D.L.); chen.hao1@zs-hospital.sh.cn (H.C.); sun.jie3@zs-hospital.sh.cn (J.S.); li.haojie@zs-hospital.sh.cn (H.L.); sun.yihong@zs-hospital.sh.cn (Y.S.); 2 Gastric Cancer Center, Zhongshan Hospital, Fudan University, Shanghai 200032, China; 3 Department of General Surgery, Zhongshan Hospital (Xiamen), Fudan University, Xiamen 361000, China; liu.xinyou@zsxmhospital.com; 4 Xiamen Clinical Research Center for Cancer Therapy, Xiamen 361000, China

**Keywords:** gastric cancer, lymphovascular invasion, prognosis, recurrence pattern

## Abstract

**Background and Aim:** Lymphovascular invasion (LVI) is a negative prognostic factor for gastric cancer, but detection limitations hinder its clinical utility and subtype analysis. This study aimed to explore the predictive value of LVI and its subtypes in the prognosis and recurrence patterns of gastric cancer using our enhanced detection method. **Methods:** We reviewed 2057 patients who underwent gastrectomy in 2018, of whom 1073 met the inclusion criteria. Propensity score matching (PSM) was performed to balance baseline clinicopathological characteristics. **Results:** After PSM, 311 patients were assigned to the LVI+ group and 311 to the LVI- group. The LVI+ group demonstrated a poorer prognosis. Subtype analysis revealed that lymphatic invasion (LI), but not venous invasion (VI), was associated with poor prognosis in the matched cohort. Stratified by pathological tumor-node-metastasis (TNM) stage, LVI+ and LI+ patients had worse prognosis in Stages I and III, while VI+ patients had worse prognosis in Stage III. Stratified by lymph node status, LVI+ predicted poorer prognosis in both node-negative (N0) and node-positive (N+) patients, and LI+ was also associated with worse prognosis among N+ patients, whereas VI+ was not significantly associated with prognosis in either subgroup. Recurrence analysis indicated that LVI+ was associated with distant and peritoneal metastases, whereas LI+ was associated with local recurrence, distant and peritoneal metastases. **Conclusions:** Lymphovascular invasion was associated with adverse prognosis in resectable gastric cancer, with lymphatic invasion showing a stronger prognostic impact than venous invasion. These findings indicate that refined assessment of lymphovascular invasion may complement conventional TNM staging in postoperative risk stratification.

## Simple Summary

Lymphovascular invasion indicates aggressive gastric cancer, but conventional detection methods often lack sensitivity. The aim of our large-scale retrospective study was to reassess the prognostic value of this invasion and its specific subtypes using an enhanced detection method. Evaluating a cohort of over 1000 patients who underwent curative gastrectomy, we demonstrated that lymphatic invasion independently predicts poorer survival and higher risks of local recurrence and peritoneal metastasis, whereas venous invasion was not independently associated with overall survival in the matched cohort. Accurately discriminating lymphatic from venous invasion provided valuable additional information, clarifying which patients remain at high risk despite otherwise favorable traditional staging. Therefore, this enhanced pathology-based marker is highly useful for post-operative risk stratification and, alongside standard staging, provides essential evidence to support more personalized decisions regarding adjuvant treatment intensity and surveillance.

## 1. Introduction

Gastric cancer (GC) remains a major global health burden, particularly in East Asia [1,2]. GC development is driven primarily by Helicobacter pylori infection and is further shaped by smoking, high-salt dietary exposure, obesity, Epstein–Barr virus-associated disease, and hereditary susceptibility; malignant transformation commonly follows progression from chronic gastritis to atrophy, intestinal metaplasia, dysplasia, and invasive carcinoma [3,4,5,6]. Accordingly, prevention and early interception rely on H. pylori eradication and surveillance of high-risk precursor lesions [3,4]. Therapeutic management is increasingly multimodal, ranging from endoscopic resection and radical gastrectomy with lymphadenectomy to perioperative or adjuvant therapy, whereas biomarker-guided targeted therapy and immunotherapy have further expanded options for advanced disease [7,8,9,10,11,12]. However, for resectable disease, the role of postoperative radiotherapy remains unsettled, and major gastric-cancer trials, including ARTIST, CRITICS, and ARTIST 2, have not established a uniform benefit of adjuvant chemoradiotherapy after adequate surgery [10,11,12]. Nevertheless, patients with the same pathological tumor-node-metastasis (TNM) stage may still experience markedly different outcomes, suggesting that conventional staging alone does not fully capture the biological aggressiveness of GC [13,14,15].

Metastatic spread in GC is not uniform. Tumor dissemination may proceed through lymphatic, hematogenous, and peritoneal routes, which are associated with distinct recurrence patterns and clinical consequences [15,16,17,18,19]. At the molecular level, GC invasion is closely linked to lymphangiogenesis, extracellular matrix remodeling, and adhesion-related signaling. Common invasion-related pathways include VEGF-C/VEGF-D-VEGFR-3 signaling, MMP-2-mediated stromal remodeling, PDGFRα-associated stromal activation, and transforming growth factor-β-mediated peritoneal adhesion [16,17,18,20]. Postoperative follow-up is also evolving from imaging and conventional serum markers alone toward molecular, cellular, and liquid-biopsy surveillance; in particular, circulating tumor DNA (ctDNA) and circulating tumor cells (CTCs) are increasingly being explored for minimal residual disease assessment, recurrence prediction, and dynamic monitoring after treatment [21,22].

Within this context, lymphovascular invasion (LVI), defined as the presence of malignant cells within lymphatic or vascular channels, represents an early morphological event in the invasion-metastasis cascade [15,20]. Growing evidence suggests that LVI is associated with tumor invasion, nodal spread, recurrence, and poor prognosis in GC [23,24,25,26,27,28,29,30,31]. However, its clinical application remains limited, and the distinct clinical implications of its two subtypes, lymphatic invasion (LI) and venous invasion (VI), remain insufficiently clarified [32,33,34,35,36,37,38,39]. One important reason may be the limited sensitivity of conventional hematoxylin and eosin staining and its inability to reliably distinguish LI from VI [15,36]. This broader move toward metastasis-oriented risk stratification is consistent with recent 2026 work showing that dedicated prediction models can improve estimation of lymph node metastasis and long-term outcome beyond conventional clinicopathologic assessment in early gastric cancer [40].

Using our enhanced immunohistochemistry-based method, which improves both LVI detection and subtype discrimination [15], we hypothesized that a more accurate assessment of LVI and its subtypes would better capture route-specific metastatic behavior and provide prognostic information beyond conventional staging. We therefore aimed to reassess the associations of LVI, LI, and VI with survival and recurrence patterns in resectable GC, and to clarify their value for postoperative risk stratification, follow-up planning, and multimodal treatment selection.

## 2. Materials and Methods

### 2.1. Patients

Patients with gastric cancer who underwent gastrectomy at our hospital between January and December 2018 were enrolled in a prospective database.

The inclusion criteria were as follows: (1) pathological diagnosis of gastric adenocarcinoma; (2) no neoadjuvant treatment before operation; (3) underwent curative gastrectomy with standard lymphadenectomy; (4) R0 resection (negative resection margins); (5) underwent lymphovascular invasion assessment using D2-40 immunohistochemistry (clone D2-40, monoclonal mouse anti-human podoplanin; Agilent Technologies, Santa Clara, CA, USA) and standard in-house Elastica van Gieson (EVG) staining; (6) TNM stage I-III according to the criteria of the American Joint Committee on Cancer TNM staging system (version 8) [41].

The exclusion criteria included: (1) gastric remnant carcinoma; (2) patients with other concurrent malignant tumors; (3) TNM stage IV; (4) pathological diagnosis of nonadenocarcinoma after surgery; (5) patients with incomplete clinicopathological or follow-up data.

Patient demographic and baseline clinicopathological characteristics are summarized in Table 1.

The study was reviewed and approved by the Institutional Review Board (IRB) of our hospital (NO. B2018-186(2)), and the study was conducted in accordance with the Helsinki Declaration of 1964 and later versions. Autonomous, non-coerced consent was obtained from donors/representatives, and no organs/tissues were sourced from executed or conscience prisoners. Informed consent was obtained from all patients.

### 2.2. Pathological Assessment

Our previous research and preliminary preparations, involving more than 800 cases of enhanced LVI detection method using IHC staining, have refined the method to be robust and reproducible, and led to the establishment of a unified diagnostic standard [15]. To ensure objectivity, all specimens were independently reviewed by two experienced pathologists who were not involved in any part of the data analysis. When discrepant assessments of LVI, LI, or VI occurred, the slides were submitted to a third pathologist for final adjudication.

### 2.3. Follow-Up

Patients were followed every 3 months after surgery, primarily by telephone, until August 2022. Telephone follow-up was used to ascertain survival status and to collect information on major clinical events and relevant hospital visits. Overall survival (OS) was defined as the interval from the date of surgery to death from any cause. For patients who died, the date of death was determined preferentially from hospital records or other available medical documentation. When death occurred outside our institution and documentary confirmation was unavailable, the date reported by family members during follow-up was used. Patients who were alive at the end of follow-up were censored on the last date on which survival status was confirmed. When the exact date of death could not be verified but the month of death was known, the 15th day of that month was assigned.

The median follow-up duration was 48 months (range, 4.8–52.8 months). Because follow-up was closed in August 2022 and the median follow-up was 48 months, mature 5-year OS data were not yet available for the full cohort; therefore, 4-year OS was reported as the most informative fixed-time survival estimate, whereas the primary prognostic assessment was based on Kaplan–Meier and Cox analyses using all available follow-up data. The follow-up rate was 84.6%. Among the included patients, those who were alive at the end of follow-up were censored on the last date on which survival status was confirmed. A total of 195 patients were lost to follow-up before an analyzable survival endpoint could be ascertained and were therefore excluded from the present analysis; thus, 1073 patients with analyzable follow-up data were included.

### 2.4. Propensity Score Matching (PSM)

The Pathological TNM stage was significantly different among groups in initial analysis, which made it impossible to distinguish whether the difference in prognosis is due to LVI or pathological stage. Therefore, propensity score matching was conducted to control for imbalances in baseline clinicopathological characteristics. A 1:1 PSM analysis (without replacement) was conducted via the nearest neighbor method with a caliper value of 0.01. After matching, groups were considered well-balanced when the Standardized Mean Difference (SMD) < 0.1 and *p* > 0.05.

### 2.5. Statistical Analysis

All analyses were performed with the IBM SPSS Statistics version 27.0 and R version 4.3.2.

OS was estimated using the Kaplan–Meier method and compared with the log-rank test. Hazard ratios (HRs) and 95% confidence intervals (CIs) were estimated using Cox proportional hazards models. Differences in recurrence patterns were assessed using Pearson’s chi-square test, the continuity-corrected chi-square test, or Fisher’s exact test, as appropriate. Odds ratios (ORs) and 95% CIs were also calculated. All tests were two-sided, and a *p* value < 0.05 was considered statistically significant.

## 3. Results

### 3.1. Patient Recruitment and Clinicopathological Features

**Figure 1:**
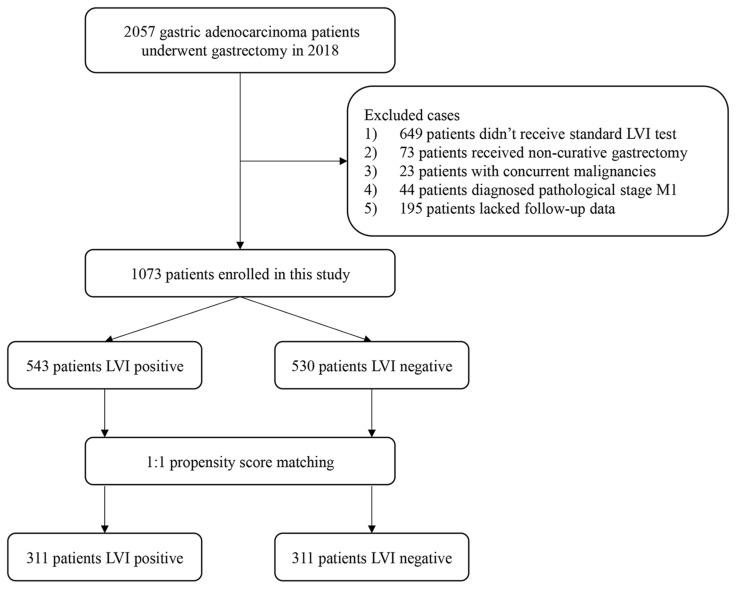
Flow chart of patient selection. A total of 2057 patients with gastric adenocarcinoma who underwent gastrectomy in 2018 were screened. After application of the inclusion and exclusion criteria, 1073 eligible patients were included in the final analysis. Among them, 543 patients were LVI-positive and 530 were LVI-negative; after 1:1 propensity score matching, 311 patients remained in each group. Abbreviation: LVI, lymphovascular invasion.

A total of 2057 patients who underwent gastrectomy at our hospital in 2018 were screened; 1073 met the inclusion criteria and were included in the analysis (Figure 1). Before PSM, baseline clinicopathological characteristics were imbalanced between the groups. Compared with the LVI− group, patients in the LVI+ group presented at a more advanced TNM stage, had larger tumor size, were more frequently classified as diffuse or mixed by Lauren type, and exhibited poorer tumor differentiation. Including these factors as matching variables. After PSM, baseline clinicopathological characteristics were substantially balanced between the groups, although a slight residual difference in tumor size remained (Table 1). Tumor size was therefore retained in the subsequent multivariable Cox analyses.

In addition to survival outcomes, we assessed the association between LVI and its subtypes and lymph node metastasis. As shown in Supplementary Figure S1, no statistically significant differences were observed between LVI+, LI+, or VI+ groups and their respective negative counterparts (all *p* > 0.05).

### 3.2. Prognosis Analysis of LVI and Its Subtypes

**Figure 2:**
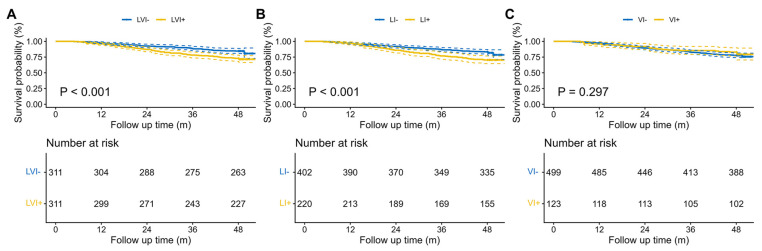
Kaplan–Meier overall survival curves according to LVI status and its subtypes. Kaplan–Meier curves of overall survival for patients stratified by LVI status (**A**), lymphatic invasion status (**B**), and venous invasion status (**C**). Survival differences were compared using the log-rank test. Numbers at risk are shown below each panel. A two-sided *p* < 0.05 was considered statistically significant. Abbreviations: LVI+, patients with lymphovascular invasion; LI+, patients with lymphatic invasion; VI+, patients with venous invasion; LVI−, patients without lymphovascular invasion; LI−, patients without lymphatic invasion; VI−, patients without venous invasion.

By the time of analysis, a total of 161 deaths (26.35%) had been recorded. The Kaplan–Meier OS curve comparing the LVI+ group and LVI− group is shown in Figure 2A. LVI+ group had a significantly worse prognosis (*p* < 0.001, HR = 1.856, 95%CI 1.306–2.639). Given the duration of follow-up in the present cohort, the 4-year OS of the LVI− group was 84.6% and 73.0% in the LVI+ group.

Kaplan–Meier curves for OS according to LI and VI status are shown in Figure 2B,C. Compared with the LI− group, the LI+ group had significantly worse OS (*p* < 0.001; HR, 1.829; 95% CI, 1.303–2.568). The 4-year OS rate was 83.3% in the LI− group and 70.5% in the LI+ group. By contrast, VI status was not significantly associated with OS (*p* = 0.3). The prognostic value of LVI and LI was further supported by the univariable and multivariable Cox analyses (Supplementary Tables S1–S3).

### 3.3. Prognostic Impact of LVI and Its Subtypes Stratified by TNM Stage

**Figure 3:**
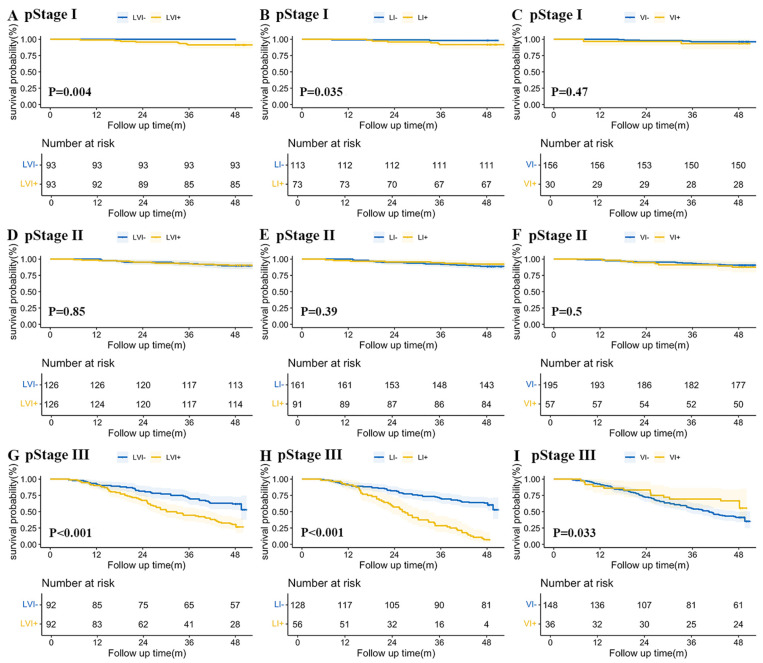
Kaplan–Meier overall survival curves according to LVI status and its subtypes stratified by TNM stage. Panels (**A**–**C**) represent stage I disease, panels (**D**–**F**) represent stage II disease, and panels (**G**–**I**) represent stage III disease. Within each stage, survival differences were compared using the log-rank test. Numbers at risk are shown below each panel. A two-sided *p* < 0.05 was considered statistically significant. Abbreviations: LVI+, patients with lymphovascular invasion; LI+, patients with lymphatic invasion; VI+, patients with venous invasion; LVI−, patients without lymphovascular invasion; LI−, patients without lymphatic invasion; VI−, patients without venous invasion; pStage, pathologic TNM stage.

Kaplan–Meier overall survival curves for patients with LVI and its subtypes by TNM stage are shown in Figure 3. For TNM Stage I patients, both the LVI+ (*p* = 0.004) and LI+ (*p* = 0.035) groups had significantly poorer prognosis, whereas no significant difference was observed for VI. No significant differences were observed in TNM Stage II for LVI, LI, or VI. For TNM Stage III patients, the LVI+ group (*p* < 0.001), LI+ group (*p* < 0.001), and VI+ group (*p* = 0.033) all had a significantly worse prognosis.

### 3.4. Prognostic Significance of LVI and Its Subtypes Stratified by Lymph Node Status

**Figure 4:**
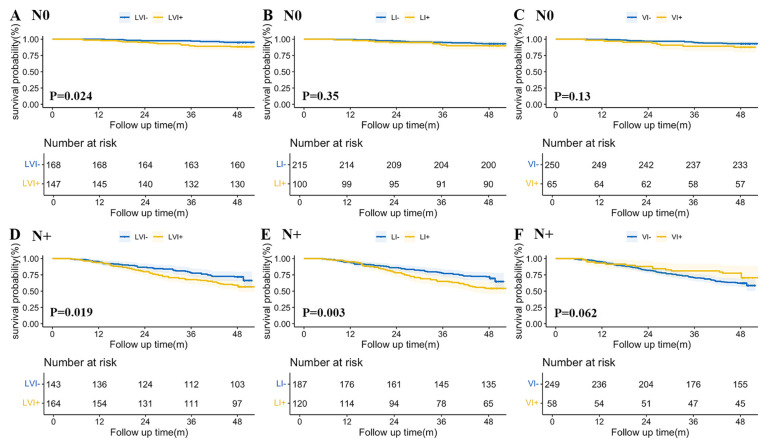
Kaplan–Meier overall survival curves according to LVI status and its subtypes stratified by lymph node status. Panels (**A**–**C**) represent node-negative disease, and panels (**D**–**F**) represent node-positive disease. Survival differences were compared using the log-rank test. Numbers at risk are shown below each panel. A two-sided *p* < 0.05 was considered statistically significant. Abbreviations: LVI+, patients with lymphovascular invasion; LI+, patients with lymphatic invasion; VI+, patients with venous invasion; LVI−, patients without lymphovascular invasion; LI−, patients without lymphatic invasion; VI−, patients without venous invasion; N0, node-negative; N+, node-positive.

Kaplan–Meier OS curves for LVI and its subtypes according to lymph node status are shown in Figure 4. For node-negative (N0) patients, although the LVI+ group showed significantly poorer survival (*p* = 0.02, HR = 2.537, 95%CI 1.095–5.879), the prognostic significance of LI+ (*p* = 0.35) and VI+ (*p* = 0.13) was not statistically evident. For node-positive (N+) patients, the LVI+ group (*p* = 0.02, HR = 1.584, 95%CI 1.075–2.355) and the LI+ group (*p* = 0.003, HR = 1.746, 95%CI 1.199–2.542) demonstrated significantly worse prognosis, whereas VI+ did not reach statistical significance (*p* = 0.062).

### 3.5. Analysis of Recurrence Patterns According to LVI and Its Subtypes

Table 2 shows the distribution of local recurrence, distant metastasis, and peritoneal metastasis according to LVI status and its subtypes. A total of 215 recurrences and metastases occurred in 112 patients (18.0%), with their specific sites and corresponding proportions detailed in Supplementary Table S4.

Compared to the LVI- group, the LVI+ group had a significantly higher proportion of distant metastasis (*p* = 0.03, OR = 1.670, 95%CI 1.039–2.685) and peritoneal metastasis (*p* = 0.03, OR = 3.764, 95%CI 1.040–13.627). In the subtype analysis, the LI+ group had a significantly higher proportion of local recurrence (*p* = 0.01, OR = 2.439, 95%CI 1.213–4.902) and peritoneal metastasis (*p* = 0.01, OR = 4.738, 95%CI 1.468–15.289), whereas no statistically significant differences were observed in the recurrence patterns of the VI+ group.

LVI and lymph node metastasis had synergistic effects not only on prognosis but also on recurrence patterns. Compared with other patients, the LVI+ & N+ group showed a significantly higher tendency for local recurrence (*p* < 0.001), distant metastasis (*p* < 0.001), and peritoneal metastasis (*p* = 0.02), as detailed in Supplementary Table S5.

## 4. Discussion

Gastric cancer remains a major global health burden, particularly in East Asia [1,2], yet outcomes are not fully captured by conventional TNM staging alone. In this context, lymphovascular invasion may provide additional biological information because it reflects an early morphologic step in tumor dissemination rather than simply tumor extent at resection. Using our enhanced detection strategy, we found that LVI, particularly lymphatic invasion (LI), was associated with adverse survival and distinct recurrence patterns. This result is in line with previous studies showing that LVI remains prognostically relevant even in node-negative gastric cancer [23,31].

The predominance of LI over venous invasion (VI) in our cohort is biologically plausible. Compared with blood vessels, lymphatic channels are structurally more permissive to tumor-cell entry, and gastric cancer commonly spreads through early lymphatic permeation and nodal dissemination [16]. At the same time, our findings should not be interpreted as excluding a role for VI. Some previous studies, especially in more advanced disease, have reported that VI is associated with poorer survival and may be more closely related to hematogenous recurrence [37,42]. Taken together, these data suggest that LI may dominate the broader resectable setting, whereas VI may be particularly relevant in selected high-risk subgroups.

Regarding the principal metastatic route of gastric cancer, no single pathway explains all patients. Nevertheless, in resectable disease, the lymphatic route appears to be the most common early track, while peritoneal spread represents a distinct transcoelomic process driven by serosal exposure, free tumor-cell shedding, and implantation in a permissive peritoneal niche [17,43,44]. This framework is consistent with our observation that LVI was associated with distant and peritoneal recurrence, whereas LI also correlated with local recurrence.

Several host and environmental factors also merit discussion. Smoking is among the most consistent risk-related exposures in gastric cancer and has additionally been linked to poorer survival in large contemporary cohorts [45]. By contrast, the effect of alcohol appears less uniform, although a large Japanese cohort reported a dose-related increase in gastric-cancer risk among men [46]. Dietary exposure is also complex: processed meat and nitrosamine-related preservatives have been implicated in gastric carcinogenesis, whereas prospective evidence for nitrate/nitrite exposure has not been entirely consistent. Water pollution may also contribute to high-incidence areas, but the available evidence is still largely ecological and should therefore be interpreted cautiously [47,48]. Sex and body weight are likewise relevant, as recent multicenter data suggest that women have slightly better survival than men, while smoking burden and BMI remain prognostically informative [45].

Among emerging biomarkers, the most clinically promising in the near term are those that can be integrated into postoperative risk stratification. First, subtype-resolved LVI assessed with improved pathology remains a practical tissue biomarker [15]. Second, stromal and extracellular-matrix features, including collagen-based signatures, may help refine metastatic risk beyond routine histology [13]. Third, liquid biopsy is increasingly relevant, and recent evidence indicates that postoperative ctDNA can predict recurrence and survival after curative-intent treatment for resectable gastric or gastroesophageal adenocarcinoma [49]. Cellular biomarkers should also be acknowledged, as circulating tumor cells (CTCs) have shown value for postoperative recurrence prediction in gastric cancer cohorts [22]. Beyond tissue and liquid-biomarker approaches, recent 2026 work further suggests that metastasis-oriented prediction is becoming clinically actionable; a multimodal digital-biopsy model accurately predicted occult peritoneal metastasis before surgery and outperformed single-modality assessment [50]. In our view, a combined framework incorporating LI/VI-aware pathology and selected molecular markers may ultimately offer more precise risk assessment than TNM staging alone.

The novelty of the present study lies in reassessing LVI with an enhanced method that improves both detection and subtype classification, rather than relying on conventional evaluation alone [15]. This is important because under-recognition and subtype misclassification may partly account for inconsistent conclusions in the previous literature. In addition, we examined not only survival but also recurrence patterns in a propensity-score-matched cohort, which provides a more clinically interpretable view of metastatic behavior. Several limitations should also be acknowledged. This was a retrospective single-center study, and residual confounding cannot be fully excluded despite matching, and follow-up was not sufficiently mature for all patients. Moreover, recurrence data were not granular enough to support formal route-specific time-to-event modeling, and no external validation or integrated molecular testing was available.

However, our findings should not be interpreted as supporting adjuvant radiotherapy for any LVI-defined subgroup. In resectable gastric cancer, major gastric-cancer-specific trials, including ARTIST, CRITICS, and ARTIST 2, suggest that the benefit of postoperative chemoradiotherapy is not uniform and remains context-dependent, particularly after adequate surgery such as D2 resection [10,11,12]. Therefore, although LVI—especially LI—may help refine postoperative risk stratification, whether LVI-defined subgroups derive differential benefit from radiotherapy remains unknown and warrants prospective validation.

In summary, our findings suggest that LVI in gastric cancer should not be regarded as a purely ancillary histologic feature. When assessed with an enhanced method, LVI—especially LI—captures clinically relevant metastatic biology beyond conventional staging and may improve postoperative risk stratification [15,23].

## 5. Conclusions

In this propensity score-matched cohort, lymphovascular invasion was associated with worse survival and recurrence in resectable gastric cancer. Among its subtypes, lymphatic invasion showed a stronger and more consistent prognostic impact than venous invasion. These findings suggest that refined assessment of lymphovascular invasion, particularly lymphatic invasion, may complement TNM staging in postoperative risk stratification and clinical decision-making.

## Figures and Tables

**Table 1:** Clinicopathological characteristics of the LVI-negative and LVI-positive groups before and after 1:1 propensity score matching.

Factors	Before 1:1 PSM			After 1:1 PSM		
	LVI− (*n* = 530)	LVI+ (*n* = 543)	*p* Value	LVI− (*n* = 311)	LVI+ (*n* = 311)	*p* Value
Sex			0.208			1.000
Male	362 (68.3%)	391 (72.0%)		215 (69.1%)	215 (69.1%)	
Female	168 (31.7%)	152 (28.0%)		96 (30.9%)	96 (30.9%)	
BMI (kg/m^2^)	22.9 ± 3.4	23.4 ± 11.0	0.888	22.9 ± 3.4	22.8 ± 3.3	0.765
Tumor size (cm)	3.4 ± 1.7	4.2 ± 1.9	**<0.001**	3.6 ± 1.9	3.9 ± 1.7	0.053
Histology			0.138			0.729
Adenocarcinoma	369 (69.6%)	354 (65.2%)		217 (69.8%)	212 (68.2%)	
Signet ring and mucinous cell carcinoma	161 (30.4%)	189 (34.8%)		94 (30.2%)	99 (31.8%)	
TNM stage			**<0.001**			1.000
I	312 (58.9%)	93 (17.1%)		93 (29.9%)	93 (29.9%)	
II	126 (23.8%)	131 (24.1%)		126 (40.5%)	126 (40.5%)	
III	92 (17.4%)	319 (58.7%)		92 (29.6%)	92 (29.6%)	
Lauren			**<0.001**			0.444
Intestinal	232 (43.8%)	148 (27.3%)		125 (40.2%)	111 (35.7%)	
Mixed	216 (40.8%)	290 (53.4%)		136 (43.7%)	151 (48.6%)	
Diffuse	82 (15.5%)	105 (19.3%)		50 (16.1%)	49 (15.8%)	
Differentiation			**<0.001**			0.241
Moderately and well differentiated	169 (31.9%)	73 (13.4%)		73 (23.5%)	60 (19.3%)	
Poorly differentiated	361 (68.1%)	470 (86.6%)		238 (76.5%)	251 (80.7%)	

Data are presented as *n* (%) or mean ± standard deviation (SD). Categorical variables were compared using the chi-square test or Fisher’s exact test, as appropriate. Continuous variables were compared using the independent-samples t test or Mann–Whitney U test, as appropriate. A two-sided *p* < 0.05 was considered statistically significant. Bold values indicate statistical significance. Abbreviations: LVI, lymphovascular invasion; PSM, propensity score matching; BMI, body mass index.

**Table 2:** Distribution of local recurrence, distant metastasis, and peritoneal metastasis according to LVI status and its subtypes.

Factors	LVI− (*n* = 311)	LVI+ (*n* = 311)	*p*	LI− (*n* = 402)	LI+ (*n* = 220)	*p*	VI− (*n* = 499)	VI+ (*n* = 123)	*p*
Local recurrence			0.158			**0.010**			0.099
Negative	298 (95.8%)	290 (93.2%)		387 (96.3%)	201 (91.4%)		468 (93.8%)	120 (97.6%)	
Positive	13 (4.2%)	21 (6.8%)		15 (3.7%)	19 (8.6%)		31 (6.2%)	3 (2.4%)	
Distant metastasis			**0.033**			0.083			0.949
Negative	279 (89.7%)	261 (83.9%)		356 (88.6%)	184 (83.6%)		433 (86.8%)	107 (87.0%)	
Positive	32 (10.3%)	50 (16.1%)		46 (11.4%)	36 (16.4%)		66 (13.2%)	16 (13.0%)	
Peritoneal metastasis			**0.031**			**0.010**			0.855
Negative	308 (99.0%)	300 (96.5%)		398 (99.0%)	210 (95.5%)		487 (97.6%)	121 (98.4%)	
Positive	3 (1.0%)	11 (3.5%)		4 (1.0%)	10 (4.5%)		12 (2.4%)	2 (1.6%)	

Data are presented as *n* (%). Comparisons between groups were performed using the chi-square test or Fisher’s exact test, as appropriate. A two-sided *p* < 0.05 was considered statistically significant. Bold values indicate statistical significance. Abbreviations: LVI, lymphovascular invasion; LI, lymphatic invasion; VI, venous invasion.

## Data Availability

The datasets generated during and/or analyzed during the current study are not publicly available but are available from the corresponding author on reasonable request.

## Supplementary Materials

The following supporting information can be downloaded at: https://www.mdpi.com/article/10.3390/cancers18071101/s1. Table S1. Univariate and multivariable Cox regression analyses of overall survival by Lymphovascular invasion status (after Propensity Score Matching). Table S2. Univariate and multivariable Cox regression analyses of overall survival by Lymphatic invasion status (after Propensity Score Matching). Table S3. Univariate and multivariable Cox regression analyses of overall survival by Venous invasion status (after Propensity Score Matching). Table S4. Specific sites of the recurrence patterns. Table S5. The synergistic effect of lymphovascular invasion and lymph node metastasis is associated with local recurrence, distant metastasis, and peritoneal metastasis. Figure S1. The relationship between LVI and its subtypes LI, VI and lymph node metastasis.

## Abbreviations

DFS	disease-free survival
HR	hazard ratio
LVI	lymphovascular invasion
LI	lymphatic invasion
OS	overall survival
OR	odds ratio
PSM	propensity score matching
VI	venous invasion
