# Supplementary material for: Reassessment of Lymphovascular Invasion and Its Subtypes as Predictors of Prognosis and Recurrence in Gastric Cancer Using an Enhanced Detection Method
Source: Cancers (Basel). 2026 Mar 28;18(7):1101. doi: 10.3390/cancers18071101 (PMC13072422; doi:10.3390/cancers18071101)
Supplement: Supplementary file 1 [file cancers-18-01101-s001.zip › cancers-4201707-supplementary.pdf]

Supplementary Table S1. Univariate and multivariable Cox regression analyses of overall survival by Lymphovascular invasion status (after Propensity Score Matching)

Factors	Univariate Cox regression			Multivariable Cox regression		
	HR	95% CI	p Value	HR	95% CI	p Value
Sex (female/male)	0.959	0.662-1.388	0.824			
BMI (kg/m2)	0.957	0.908-1.009	0.100			
Tumor size (cm)	1.356	1.255-1.466	**0.000**	1.178	1.072-1.295	**0.001**
Histology (Signet ring and mucinous cell carcinoma/ Adenocarcinoma)	0.899	0.619-1.306	0.577			
pTNM stage			**0.000**			**0.000**
I	1.000			1.000		
II	2.356	1.063-5.224	0.035	1.946	0.871-4.346	0.105
III	17.664	8.592-36.317	0.000	13.648	6.473-28.773	0.000
Lauren			0.057			0.306
Intestinal	1.000			1.000		
Mixed	1.245	0.842-1.839	0.272	0.813	0.544-1.213	0.311
Diffuse	1.782	1.110-2.860	**0.017**	1.128	0.692-1.840	0.629
Differentiation (Poorly differentiated / Moderately and well differentiated)	2.512	1.444-4.370	**0.001**	1.440	0.810-2.560	0.214
LVI (LVI+/ LVI-)	1.856	1.306-2.639	**0.001**	2.232	1.563-3.188	**0.000**

Abbreviations: LVI, lymphovascular invasion. PSM, propensity score matching. BMI, body mass index.

Supplementary Table S2. Univariate and multivariable Cox regression analyses of overall survival by Lymphatic invasion status (after Propensity Score Matching)

**Factors**	**Univariate Cox regression**			**Multivariable Cox regression**		
	HR	95% CI	p Value	HR	95% CI	p Value
Sex (female/male)	0.959	0.662-1.388	0.824			
BMI (kg/m2)	0.957	0.908-1.009	0.100			
Tumor size (cm)	1.356	1.255-1.466	**0.000**	1.162	1.055-1.280	**0.002**
Histology (Signet ring and mucinous cell carcinoma/ Adenocarcinoma)	0.899	0.619-1.306	0.577			
pTNM stage			**0.000**			**0.000**
I	1.000			1.000		
II	2.356	1.063-5.224	0.035	2.112	0.944-4.725	0.069
III	17.664	8.592-36.317	0.000	17.274	8.065-36.998	0.000
Lauren			0.057			0.177
Intestinal	1.000			1.000		
Mixed	1.245	0.842-1.839	0.272	0.725	0.482-1.091	0.123
Diffuse	1.782	1.110-2.860	**0.017**	1.029	0.632-1.675	0.909
Differentiation (Poorly differentiated / Moderately and well differentiated)	2.512	1.444-4.370	**0.001**	1.240	0.693-2.221	0.469
LI (LI+/ LI-)	1.829	1.303-2.568	**0.000**	2.892	2.030-4.121	**0.000**

Abbreviations: LI, lymphatic invasion. PSM, propensity score matching. BMI, body mass index.

Supplementary Table S3. Univariate and multivariable Cox regression analyses of overall survival by Venous invasion status (after Propensity Score Matching)

**Factors**	**Univariate Cox regression**			**Multivariable Cox regression**		
	HR	95% CI	p Value	HR	95% CI	p Value
Sex (female/male)	0.959	0.662-1.388	0.824			
BMI (kg/m2)	0.957	0.908-1.009	0.100			
Tumor size (cm)	1.356	1.255-1.466	**0.000**	1.167	1.066-1.278	**0.001**
Histology (Signet ring and mucinous cell carcinoma/ Adenocarcinoma)	0.899	0.619-1.306	0.577			
pTNM stage			**0.000**			**0.000**
I	1.000			1.000		
II	2.356	1.063-5.224	0.035	1.983	0.886-4.441	0.096
III	17.664	8.592-36.317	0.000	13.031	6.135-27.682	0.000
Lauren			0.057			0.485
Intestinal	1.000			1.000		
Mixed	1.245	0.842-1.839	0.272	0.826	0.550-1.242	0.358
Diffuse	1.782	1.110-2.860	**0.017**	1.047	0.638-1.718	0.856
Differentiation (Poorly differentiated / Moderately and well differentiated)	2.512	1.444-4.370	**0.001**	1.302	0.720-2.354	0.383
VI (VI+/ VI-)	0.785	0.497-1.239	0.298	0.762	0.479-1.210	0.249

Abbreviations: VI, venous invasion. PSM, propensity score matching. BMI, body mass index.

Supplementary Table S4. Specific sites of the recurrence patterns

Patterns	Patients
Local recurrence (n=34)	
Lymph nodes [a]	27 (4.3%)
Remnant stomach and Anastomosis	26 (4.2%)
Distant metastasis (n=82) [b]	
Lymph nodes [a]	51 (8.2%)
Liver	39 (6.3%)
Lung	28 (4.5%)
Bone	20 (3.2%)
Others [c]	10 (1.6%)
Peritoneal metastasis (n=14)	14 (2.3%)

a Virchow node belongs to Distant.

b Distant metastasis not includes peritoneal metastasis.

c Others include brain, intestines, pleural, kidney, ovaries and uterus.

Supplementary Table S5. The synergistic effect of lymphovascular invasion and lymph node metastasis is associated with local recurrence, distant metastasis, and peritoneal metastasis

**Factors**	Other (n=458)	LVI+ & N+ (n=164)	P
Local recurrence			**< 0.001**
Negative	442 (96.5%)	146 (89.0%)	
Positive	16 (3.5%)	18 (11.0%)	
Distant metastasis			**< 0.001**
Negative	415 (90.6%)	125 (76.2%)	
Positive	43 (9.4%)	39 (23.8%)	
Peritoneal metastasis			**0.019**
Negative	452 (98.7%)	156 (95.1%)	
Positive	6 (1.3%)	8 (4.9%)	

Abbreviations: LVI+ & N+, patients with lymphovascular invasion and lymph node metastasis. Other, patients without concurrent lymphovascular invasion and lymph node metastasis.

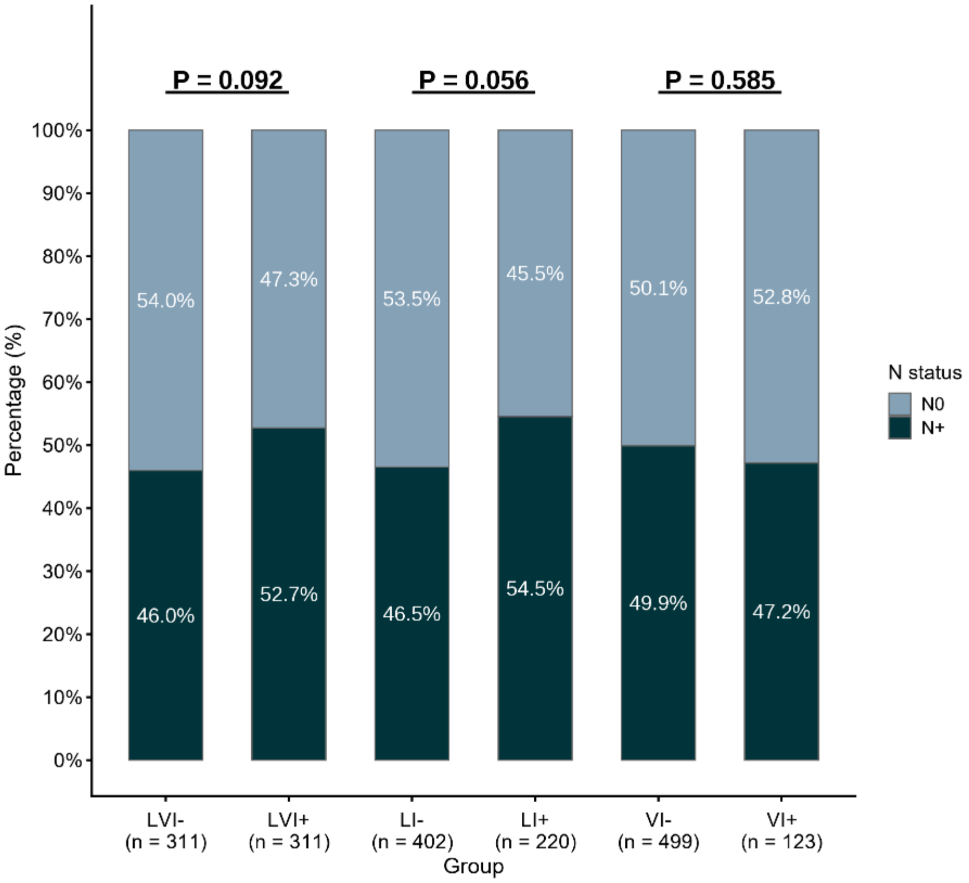

Supplementary Figure S1. The relationship between LVI and its subtypes LI, VI and lymph node metastasis.

Abbreviations: LVI+, patients with lymphovascular invasion. LI+, patients with lymphatic invasion but without venous invasion. VI+, patients with venous invasion but without lymphatic invasion. LVI-, patients without lymphovascular invasion. LI-, patients without lymphatic invasion. VI-, patients without venous invasion.
